# Milk Lactose as a Biomarker of Subclinical Mastitis in Dairy Cows

**DOI:** 10.3390/ani11061736

**Published:** 2021-06-10

**Authors:** Ramūnas Antanaitis, Vida Juozaitienė, Vesta Jonike, Walter Baumgartner, Algimantas Paulauskas

**Affiliations:** 1 Large Animal Clinic, Veterinary Academy, Lithuanian University of Health Sciences, Tilžės str 18, LT-47181 Kaunas, Lithuania; 2 Department of Biology, Faculty of Natural Sciences, Vytautas Magnus University, K. Donelaičio 58, LT-44248 Kaunas, Lithuania; vida.juozaitiene@vdu.lt (V.J.); vesta.jonike@vdu.lt (V.J.); algimantas.paulauskas@vdu.lt (A.P.); 3 University Clinic for Ruminants, University of Veterinary Medicine, Veterinaerplatz 1, A-1210 Vienna, Austria; walter.baumgartner@vetmeduni.ac.at

**Keywords:** bovine subclinical mastitis, milk lactose, mastitis pathogens, seasonal dynamics, Lithuania

## Abstract

**Simple Summary:**

Early bovine subclinical mastitis detection may improve treatment strategies and milk yield and reduce the use of antibiotics. Scientific research is therefore focusing on the identification of new and less expensive biomarkers. One of the biomarkers with the greatest potential is milk lactose. To establish whether lactose could be used as an indicator for subclinical mastitis, milk samples from seventy-two herds were screened for bovine subclinical mastitis agents. This study evaluated the prevalence of subclinical mastitis pathogens, their seasonal occurrence, and the relationship with milk lactose concentration. Milk samples were mostly found to be infected with mixed microbiota, coagulase-negative Staphylococcus and Staphylococcus aureus. Yeast, Enterococcus spp. and coliform bacteria such as Escherichia coli were found during the outdoor grazing period. Increases in somatic cell count and decreases in lactose concentration were directly related to the presence of bovine subclinical mastitis agents. These findings suggest that changes in lactose content could be tracked as a diagnostic method in subclinical mastitis prevention in cows.

**Abstract:**

Bovine subclinical mastitis can cause great harm to dairy herds because of its negative impact on milk production and quality and cow health. Improved diagnostic tools are needed to maximise the control of subclinical mastitis distribution and ensure the high quality of milk as an industrial product. Between 2015 and 2020, seventy-two dairy herds were screened for bovine subclinical mastitis causative agents to identify the relationship between seasons, lactose levels and subclinical mastitis infection. The predominant species found in the milk samples were mixed microbiota, coagulase-negative *Staphylococcus* and *Staphylococcus aureus*. Yeasts were found exclusively in autumn, while *Enterococcus* spp. and *Escherichia coli* were only found in summer and autumn. A negative correlation was detected between milk lactose and number of somatic cells in milk (−0.471; *p* < 0.001). The lactose levels in milk were closely associated with the prevalence (%) of subclinical mastitis pathogens, such as *Streptococcus agalactiae* (y = −1.8011x + 10.867, R^2^ = 0.9298), *Staph*. *aureus* (y = −3.5216x + 25.957, R^2^ = 0.8604) and other *Streptococci* (y = −0.5956x + 7.6179, R^2^ = 0.6656). These findings suggest that milk lactose may be used as a biomarker of suspected udder inflammation in modern health prevention programmes for cows to reduce the prevalence of subclinical mastitis pathogens in dairy cattle herds.

## 1. Introduction

Bovine subclinical mastitis (BSM) is a disease with a high incidence worldwide, and one of the most prevalent bovine pathologies causing the greatest losses to the dairy industry [1]. The prevalence of subclinical mastitis in dairy cattle herds depends on herd management and housing (indoor versus outdoor) systems, and on the quantity and diversity of udder pathogens [2]. These can be divided into two large groups by path of infection: contagious (from cow to cow, mainly during the milking process) and environmental (found throughout the dairy cows’ environment) [3]. As an inflammation of the mammary gland and udder tissue, BSM usually occurs as an immune response to an invasion of microorganisms, such as bacteria, yeasts, algae and viruses from the environment and/or transmitted from cow to cow (contagious transmission) [4,5,6]. However, the most common causes of subclinical mastitis are bacteria such as *Staphylococcus aureus*, *Streptococcus agalactiae* and coliform bacteria, such as *Escherichia coli* [7]. Depending on the manifestation of mastitis, bovine mastitis can be divided into two large groups: clinical and subclinical. Clinical mastitis causes visibly abnormal milk and alteration of the udder. In contrast to the clinical form, subclinical mastitis does not have visual signs, and can only be detected by means of an individual cow somatic cell count or microbiological culture analysis. In both cases the production of milk decreases to differing extents. Mastitis negatively impacts milk quality which adversely affects downstream milk processing [8,9].

Physiological changes, such as swelling or inflammation of the mammary gland or a change in milk yield, colour or consistency, can be the primary signs of mastitis. However, these signs occur only in the clinical form, therefore the most common method for diagnosing mastitis in dairy cows is to measure the somatic cell count [9]. Cows that have above 2.0 × 10^5^ somatic cells/mL are considered to be subclinically infected [10]. An increased number of somatic cells has been found to be associated with the decrease in milk lactose percentage due to changes in the homeostasis of mammary glands during mastitis infection [11,12,13]. Berglund et al. [14] observed that an increase in somatic cell count (from 3.1 × 10^4^ to 4.5 × 10^5^ somatic cells/mL) is associated with a reduced level of lactose (from 4.86 to 4.69%). Miglior et al. [10] also found that lactose had a negative relationship (−0.20) with the number of milk somatic cells.

Lactose is a disaccharide sugar that is made up of glucose and galactose molecules and is the major bovine milk solid (carbohydrates representing approximately 40% of the total solids and 50% of the fat-free solids). The synthesis and concentration of lactose in milk are mainly affected by udder health and the cow’s energy balance and metabolism [15,16,17]. Different studies have demonstrated unfavourable relationships with a negative energy balance and related metabolic disorders with subclinical mastitis in high-producing postpartum dairy cows [17,18,19]. Moreover, researchers have pointed at the widespread use of lactose in the food and pharmaceutical industries, and the fact that milk is the main source of lactose used in industry [20]. Registered lower levels of milk lactose can be used for early identification of metabolic disorders and subclinical mastitis (set at milk somatic cells ≥ 1.0 × 10^5^ cells/mL). Lactose levels in cows’ milk were positively associated with their reproductive success [21].

There are not enough data in the scientific literature on the relationship between milk lactose and the prevalence of subclinical mastitis pathogens. Therefore, the aim of this study was to evaluate the prevalence of subclinical mastitis pathogens, their seasonal occurrence and the relationship with milk lactose concentration. The hypothesis of the study is that milk lactose may be a biomarker of subclinical mastitis in dairy cows.

## 2. Materials and Methods

### 2.1. Location, Animals and Experimental Design

The research was performed in accordance with the provisions of the Law on Animal Welfare and Protection of the Republic of Lithuania. The study approval number is PK016965.

The study was carried out on 72 farms in the Lithuanian Black-and-White Breeders Association in 2015–2020. The feed ration in all farms was balanced to fit the energy and nutrient requirements of a 550–650 kg Holstein cow producing, on average, 30 kg/day of milk throughout the experimental period. At least 30% of the cows in each herd were selected. The average number of somatic cells in milk from the tested herds exceeded 200,000 cells/mL for at least three consecutive months. Cows (*n* = 5814) from the herds were selected at random by the herds’ veterinarians. Those cows were identified as having subclinical mastitis. The number of somatic cells in the milk of the selected cows corresponded to the average of the last inspection of that herd.

The milk samples were tested for somatic cell count, lactose content and mastitis pathogens. Causative agents of subclinical mastitis were detected in 88.8% (*n* = 5163) of the milk samples and the data of these cows used for further analysis. Cows (1563 first lactation cows, 1602s lactation cows and 1998 cows ≥ 3 lactation) were evaluated at the third or fourth month of lactation.

### 2.2. Measurements

Detection of subclinical mastitis-related microorganisms, evaluation of milk lactose and counting of somatic cells in milk samples from the cows were performed at the state enterprise “Pieno Tyrimai” following the methodology proposed by the National Mastitis Council [22].

Milk samples were composited for each cow into one sample (45 mL) in a tube containing a preservative based on boric acid (Merck KGaA, Darmstadt, Germany). Before taking the sample, each teat was cleaned with a tissue moistened with 70% ethanol solution. The first two to three streams of milk were discharged, and then milk samples were collected in a tube containing a preservative to study microorganism diversity. The samples were stored for no more than three days at a temperature of 4 ± 2 °C. For detection of mastitis agents, 10 µL milk samples were cultivated in a blood agar base with esculin and incubated for 24–72 h at a temperature of 37 ± 1 °C to determine the type of haemolysis. Grown colonies were assessed visually by appearance, size, colour and haemolysis zones. Potassium hydroxide and Gram coloration were then used to classify microorganisms into yeast, Gram-positive and Gram-negative and cocci or bacilli. The Gram-positive cocci were tested using catalase to discern *Staphylococcus (Staph.)* spp. and *Streptococcus (Strep.)* spp. *Staphylococcus* spp., which produces coagulase, ferments mannitol, and grows on Baird-Parker agar in black colonies with a clear halo, was considered to be *Staph. aureus*. For identification of *Streptococcus* and *Enterococcus*, hydrolysis of esculin was used. If hydrolysis of esculin occurred, bacteria were further tested with pyrrolidonyl aminopeptidase (PYR) test. Positive samples for PYR test were assumed to be *Enterococcus* and samples with negative reaction for PYR test were considered to be *Streptococcus* D serogroup. Those cultures which were considered to be *Streptococcus*, but did not hydrolysate esculin, were subjected for agglutination test to classify *Streptococcus* in A, B, C, F and G serogroups by Lancefield grouping. For Gram-negative bacilli, Drigalski and Chromocult Coliform agar were used. Colonies which grew on Drigalski and Chromocult Coliform agar and formed dark blue or purple colonies were assumed to be *Escherichia coli*. Samples in which more than one microorganism species was identified were considered to have mixed microbiota.

Total somatic cell count (SCC) of milk was determined by using a SomaScope (CA-3A4, 2004, Delta Instruments, Drachten, The Netherlands). The milk lactose assay was performed with a LactoScope^TM^ FTIR (Delta Instruments B.V., Drachten, The Netherlands) infrared mid-range meter. For this analysis, we had 45 mL of milk from each cow. The feed ration in all farms was balanced to fit the energy and nutrient requirements of a 550–650 kg Holstein dairy cow (on average 200 days in milk), producing, on average, 30 kg/day of milk throughout the experimental period.

### 2.3. Data Analysis and Statistics

Statistical characteristics of the sample (*n*)—arithmetic mean (M), standard error (SE) and 95% confidence interval for the mean (CI)—were calculated using statistical software SPSS (version 25, SPSS Inc., Chicago, IL, USA). Prior to analyses, the normality of all data recorded in the study was assessed by the Shapiro–Wilk normality test. The number of somatic cells in milk (SCC) was converted to somatic cell score, (SCS) (SCS = (log2 (SCC/100) ) + 3 [23]), to achieve a normal distribution. Descriptive statistics variables are presented as mean ± standard error of the mean (M ± SEM) and 95% confidence interval CI. Mean differences (assessed using the Tukey HSD—Honestly Significant Difference) were considered significant when the *p* value was < 0.05.

The cows were divided into six groups according to the concentration of lactose in milk: <4.00% (*n* = 492), 4.00–4.19% (*n* = 836), 4.20–4.39% (*n* = 1581), 4.40–4.59% (*n* = 1507), 4.60–4.79% (*n* = 646), and 4.80–5.00% (*n* = 101). The association between milk lactose and SCS was assessed using Pearson’s correlation. Linear regression was used to assess the relationship between milk lactose and changes in the proportions of subclinical mastitis pathogens.

The cows were classified according to the sampling season: spring (*n* = 1299), summer (*n* = 1291), autumn (*n* = 1293), and winter months (*n* = 1280) to assess the seasonality of the prevalence of subclinical mastitis pathogens and the relationship with the concentration of lactose in milk.

## 3. Results

### 3.1. The Prevalence of Subclinical Mastitis Pathogens Isolated from the Mammary Glands of Cows

**Figure 1:**
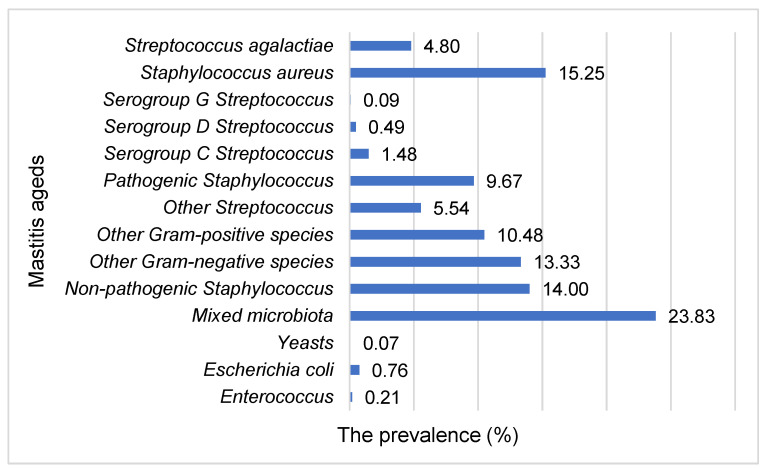
The prevalence of subclinical mastitis agents in cow’s milk samples.

Mixed microbiota, coagulase-negative *Staphylococcus* (non-pathogenic and pathogenic *Staphylococcus*) and *S aureus* were the most frequently isolated bacteria, and identified in 23.83%, 23.67% (14.00% for non-pathogenic *Staphylococcus* and 9.67% for pathogenic *Staphylococcus*) and 15.25% of samples, respectively (Figure 1). Yeasts were identified in 0.07% of samples.

**Figure 2:**
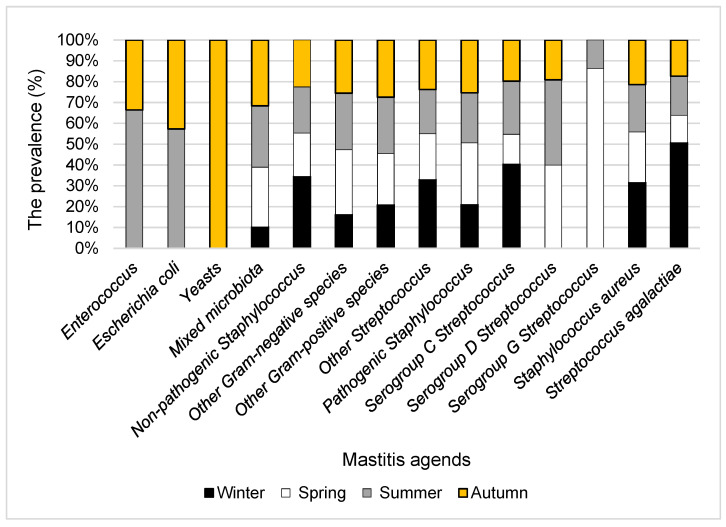
The prevalence of subclinical mastitis agents in cow’s milk samples by season.

The prevalence of subclinical mastitis agents by season is shown in Figure 2. Yeasts were only found in autumn. *Enterococcus* spp. and *E. coli* were only cultivated in summer and autumn, with a greater prevalence in summer. *Streptococcus*, which belongs to serogroup G, was detected in spring and summer. A greater prevalence of *Streptococcus* serogroup G was found in spring than in summer. Other subclinical mastitis agents with differing frequencies were identified throughout the year.

**Figure 3:**
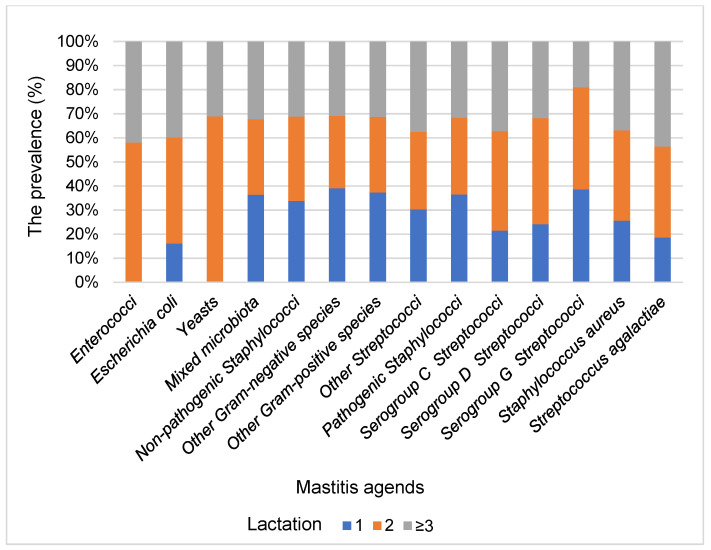
Frequency of mastitis pathogens by lactation.

In milk samples of primiparous cows, pathogens of mixed microbiota (26.49%), other Gram-negative species (16.16%) and non-pathogenic staphylococci (14.25%) prevailed. In multiparous cows (lactation ≥ 3), in addition to the aforementioned pathogens which were found in 49.39% of the samples, a higher proportion (total 16.17%) of *Staphylococcus aureus* was found (Figure 3).

### 3.2. Relationship between Milk Lactose and the Prevalence of Subclinical Mastitis Pathogens and Somatic Cell Count in Cow’s Milk

On average, the lactose content in milk was 4.35 ± 0.004%: in cows of the first parity—4.38 ± 0.007% (95% CI: 4.367–4.401%), in the second parity—4.34 ± 0.007% (95% CI: 4.324–4.361%), in cows ≥3 parities—4.32 ± 0.006% (95% CI: 4.311–4.342%).

Lactose concentrations during the winter, spring, summer and autumn were 4.29 ± 0.039% (95% CI: 4.208–4.362%), 4.37 ± 0.013% (95% CI: 4.342–4.394%), 4.33 ± 0.005% (95% CI: 4.323–4.344%) and 4.36 ± 0.005% (95% CI: 4.345–4.366%), respectively. The average somatic cell count in all tested milk samples was 701 ± 21 × 10^3^ cells/mL (95% CI: 660–742 × 10^3^ cells/mL). The largest number of somatic cells was found in the milk of tested cows in spring (1601.19 ± 29.512 × 10^3^ cells/mL), and the lowest in winter (578.47 ± 29.347 × 10^3^ cells/mL) (*p* < 0.001). The SCC of cow’s milk in summer and autumn was in the range of 589.48–650.40 × 10^3^ cells/mL.

The SCC of milk, like milk lactose, tended to increase with increasing lactation of the cows. The average SCC value in the milk of cows of the first parity (442.5 ± 44.1 × 10^3^ cells/mL, 95% CI: 365–540 × 10^3^ cells/mL) was 1.87 times lower compared to cows of the second parity and 1.91 times lower compared to ≥3 parities (*p* < 0.001).

**Figure 4:**
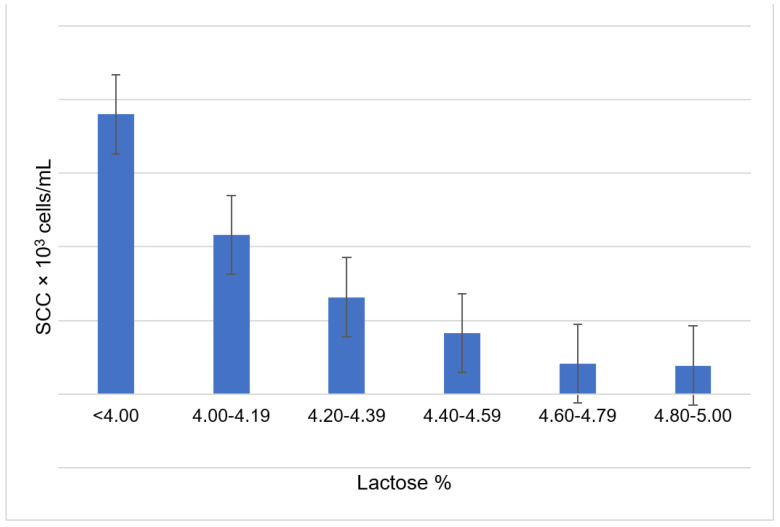
Relationship between the number of somatic cells and lactose content in milk. SCC—the number of somatic cells in milk × 10^3^ cells/mL.

A negative Pearson correlation was found between milk lactose content and SCS (−0.471; *p* < 0.001)**.** An increase in the lactose class (Figure 4) confirms a negative linear relationship with milk SCC (*p* < 0.001).

Data on the concentration of lactose in cow’s milk after the detection of various causative agents of subclinical mastitis are presented in Table 1. Analyses revealed that the lowest lactose content was found in the milk of cows infected with *S*. *agalactiae* and *S*. *aureus* (4.25–4.30%), and the highest lactose content was after the detection of serogroup G *Streptococcus* (4.55 ± 0.118%) (*p* < 0.05).

**Figure 5:**
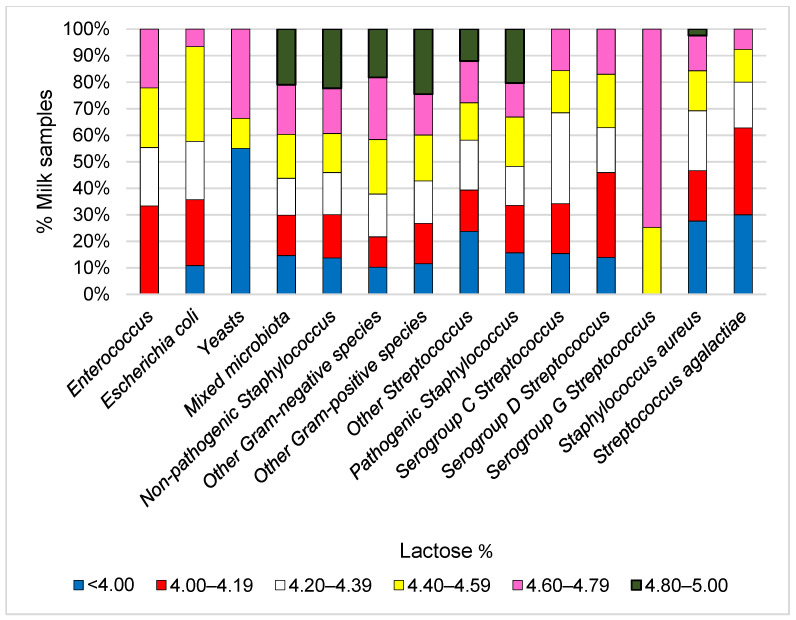
Prevalence of subclinical mastitis pathogens in milk samples based on milk lactose content.

The increase in lactose levels in cow’s milk was most closely associated with a decrease in the prevalence (%) of subclinical mastitis pathogens such as *S. agalactiae* (y = −1.8011x + 10.867, R^2^ = 0.9298), *S*. *aureus* (y = −3.5216x + 25.957, R^2^ = 0.8604), and other Streptococci (y = −0.5956x + 7.6179, R^2^ = 0.6656) (Figure 5). A substantial decrease in lactose levels was also observed in milk samples with yeast and serogroup D infection. However, due to a small number of samples, no significant differences were noticed.

## 4. Discussion

The occurrence of subclinical mastitis on dairy farms is affected by many factors. In addition to mechanical udder injuries, mastitis pathogens could be transmitted during the milking process or acquired from the environment [24,25]. In this study, almost all the microorganisms detected belonged to environmental subclinical mastitis pathogens: *E. coli*, yeasts, C, D, G group *Streptococcus* and other Streptococci. Contagious *S*. *agalactiae*, *S*. *aureus* and coagulase-negative *Staphylococcus* were also isolated and identified. However, contagious pathogens were more frequently isolated than environmental pathogens, indicating that contagious transmission may play a dominant role in the occurrence of infection. These microorganisms have also been isolated in previous studies undertaken in Lithuania [26,27], and the present results agree with the data reported by other authors related to subclinical mastitis pathogen diversity in the Baltic region [28,29].

The greatest diversity in subclinical mastitis agents was found during summer and autumn. Other studies have also shown that among the seasons, summer is the most critical time for the occurrence of subclinical mastitis pathogens in milk samples [30,31]. These results may also be related to the feeding and housing management systems maintained during the different seasons. A clear distinction could be made between cows living outdoors and those living indoors [32].

Yeasts were only detected in autumn. Similar results have been obtained in Japan [33]. Senda et al. [33] found that yeasts in milk samples were only present in summer and autumn. Moreover, four major pathogenic yeast species causing subclinical mastitis have only been detected in summer [33]. The incidence of subclinical mastitis caused by yeast is usually low in dairy herds [31,34], but large outbreaks and deadly cases have been reported [35]. Yeast mastitis has been reported in herds from environments with poor hygiene or is associated with prior antibiotic treatment of bacterial mastitis [36]. Yeasts are naturally found in moist places that are rich in organic matter and are easily isolated from teats and milking equipment [37]. Nevertheless, several species of yeast and yeast-like microorganisms have been associated with mastitis [38].

Bovine subclinical mastitis infections caused by environmental exposure to *Enterococcus* and *E. coli* were observed in summer and autumn when cows are mostly grazing outdoors. Doyle et al. [32] also noticed that during the outdoor season milk was more likely to contain higher proportions of environmental bacteria. Low amounts of *E. coli* are usually present in milk. Nonetheless, a high amount of these bacteria causes spoilage of dairy products and is an indicator of faecal contamination in the environment [39]. Moreover, if there is a large bacterial presence, it can cause endotoxic shock in cows [40]. *Enterococcus* belongs to normal gut microflora in animals and humans [39], but their pathogenic potential to cause infections of the mammary gland has been proven experimentally [41] and has been attributed to environmental mastitis agents [2].

Several studies have shown that lactose could be a potential health indicator in cows [42,43,44,45,46]. Costa et al. [17] found that cows with a lactose content of ≤4.553% had a higher rate of health impairment compared with cows with a lactose content of ≥5.045%. The authors also found that subclinical mastitis is genetically correlated with milk lactose (r = 0.518), and that more productive cows are genetically more susceptible to mastitis than less productive cows.

In cow’s milk, lactose levels vary less than fat or protein levels [10,47], thus any unexpected change in lactose content could be associated with a negative energy balance or other conditions of poor health. Changes in lactose percentage can occur due to damage in secretory cells caused by inflammation and infection and the use of milk as a substrate for growing subclinical mastitis pathogens [17]. Moreover, the amount of water activity is directly related to lactose percentage, because it osmotically determines water absorption from the cell cytosol and blood [17].

In general, the lactose contents in this study were normal and fit the ranges indicated in the literature that, according to different authors, range from 4.28 to 4.70% [10,42,47,48,49,50]. Despite this, lactose content has been related to the presence of somatic cells and subclinical mastitis pathogens in milk samples. The greatest loss of lactose content was observed in milk samples where *S*. *agalactiae*, *S*. *aureus* and other *Streptococcus* strains were detected. Bobbo et al. [51] found that milk produced by cows infected with mastitis pathogens had a lower lactose content, but statistical differences between contagious, environmental and opportunistic mastitis pathogens were not observed. A lower lactose content was also described in milk with coagulase-negative *Staphylococcus* [52,53], *S*. *aureus* [52,53], coliform bacteria [52,53] and fungal [53] infections. A great decrease in lactose content was also observed in milk samples with yeast infection. Until today, there are no data on decreases in lactose during yeast mastitis, but some yeasts, such as *Candida kefir*, are known to be able to ferment lactose to alcohol [54]. Cows with a higher lactose concentration (≥4.70%) were registered as more active and were at less risk of mastitis and metabolic disorders. Cows with higher milk lactose concentration also have a higher possibility of successful conception [21].

## 5. Conclusions

All the identified subclinical mastitis agents were found throughout the year, except for yeasts, *Enterococcus* and *E. coli*. Yeasts were isolated exclusively in autumn, while *Enterococcus* and *E. coli* were detected in summer and autumn. An increase in lactose content showed a strong negative linear relationship with SCC log10. The increase in lactose levels in cow’s milk was most closely associated with a decrease in the prevalence of subclinical mastitis pathogens such as *S*. *agalactiae*, *S*. *aureus* and other *Streptococci.* These findings underline the importance of lactose as a potential biomarker in the diagnosis of bovine mastitis. In controlled herds, this indicator could supplement information on suspected udder inflammation in cows in modern subclinical mastitis prevention programmes.

## Figures and Tables

**Table 1:** Milk lactose (%) by isolated pathogens of subclinical mastitis.

Item	Pathogens	Lactose %		95% CI	
		M	SEM	Lower Bound	Upper Bound
1	*Enterococcus* spp.	4.37 ^13^	0.079	4.214	4.523
2	*Escherichia coli*	4.36	0.041	4.284	4.445
3	Yeasts	4.32	0.137	4.056	4.591
4	Mixed microbiota	4.36 ^6,8,13,14^	0.007	4.349	4.378
5	Non-pathogenic *Staphylococcus*	4.35 ^6^	0.01	4.33	4.368
6	Other Gram-negative species	4.40 ^4,5,10,13,14^	0.01	4.379	4.417
7	Other Gram-positive species	4.36 ^13,14^	0.011	4.337	4.381
8	Other *Streptococcus*	4.32 ^4,14^	0.015	4.293	4.353
9	Pathogenic *Staphylococcus*	4.35 ^13,14^	0.012	4.325	4.37
10	Serogroup C *Streptococcus*	4.31 ^6,14^	0.03	4.255	4.371
11	Serogroup D *Streptococcus*	4.33	0.052	4.23	4.433
12	Serogroup G *Streptococcus*	4.55 ^13,14^	0.118	4.313	4.777
13	*Staphylococcus aureus*	4.30 ^4,5,6,7,9,14^	0.009	4.282	4.318
14	*Streptococcus agalactiae*	4.25 ^1,4,5,6,7,8,9,12,14^	0.016	4.215	4.279

M—mean; SEM—standard of error of the mean; 95% CI—95% confidence interval of the mean. The superscript indicates subclinical mastitis agents (number of rows in the table) with which the difference in the arithmetic mean of milk lactose was statistically significant.

## Data Availability

The data presented in this study are available within the article.

## References

[1] Gomes F., Saavedra M.J., Henriques M. (2016). Bovine mastitis disease/pathogenicity: Evidence of the potential role of microbial biofilms. Pathog. Dis..

[2] Lundberg Å. (2015). Mastitis in Dairy Cows.

[3] Hogan J.S., Martin M. (2016). Current Concepts of Bovine Mastitis.

[4] Halasa T., Huijps K., Østerås O., Hogeveen H. (2007). Economic effects of bovine mastitis and mastitis management: A review. Vet. Q..

[5] Zadoks R.N., Middleton J.R., McDougall S., Katholm J., Schukken Y.H. (2011). Molecular epidemiology of mastitis pathogens of dairy cattle and comparative relevance to humans. J. Mammary Gland Biol. Neoplasia.

[6] Jansen J., Wessels R.J., Lam T.J.G.M. January. Understanding the mastitis mindset: Applying social psychology in practice. Proceedings of the National Mastitis Council 55th Annual Meeting.

[7] Carrillo-Casas E.M., Miranda-Morales R.E. (2012). Bovine mastitis pathogens: Prevalence and effects on somatic cell count. Milk Production—An up-to-Date Overview of Animal Nutrition, Management and Health.

[8] Hameed K.G.A., Sender G., Korwin-Kossakowska A. (2007). Public health hazard due to mastitis in dairy cows. Anim. Sci. Pap. Rep..

[9] Ashraf A., Imran M. (2020). Causes, types, etiological agents, prevalence, diagnosis, treatment, prevention, effects on human health and future aspects of bovine mastitis. Anim. Health Res. Rev..

[10] Miglior F., Sewalem A., Jamprozik J., Bohmanova J., Lefebvre D.M., Moore R.K. (2007). Genetic analysis of milk urea nitrogen and lactose and their relationships with other production traits in Canadian Holstein cattle. J. Dairy Sci..

[11] Moussaoui F., Vangroenweghe F., Haddadi K., Le Roux Y., Laurent F., Duchateau L., Burvenich C. (2004). Proteolysis in milk during experimental *Escherichia coli* mastitis. J. Dairy Sci..

[12] Chedly H.B., Lacasse P., Marnet P.G., Wiart-Letort S., Finot L., Boutinaud M. (2009). Cell junction disruption after 36h milk accumulation was associated with changes in mammary secretory tissue activity and dynamics in lactating dairy goats. J. Physiol. Pharmacol..

[13] Pessora R.B., Blagitz M.G., Batista C.F., Santos B.P., Parra A.C., Souza F.N., Della Libera A.M.M.P. (2012). Avaliação da apoptose de leucócitos polimorfonucleares CH138+ em leite bovino de alta e baixa contagem de células somáticas dados preliminares. Arq. Bras. Med. Vet. Zootec..

[14] Berglund I., Pettersson G., Östensson K., Svennersten-Sjaunja K. (2007). Quarter milking for improved detection of increased SCC. Reprod. Domest. Anim..

[15] Pollott G.E. (2004). Deconstructing milk yield and composition during lactation using biologically based lactation models. J. Dairy Sci..

[16] Lemosquet S., Delamaire E., Lapierre H., Blum J.W., Peyraud J.L. (2009). Effects of glucose, propionic acid, and nonessential amino acids on glucose metabolism and milk yield in Holstein dairy cows. J. Dairy Sci..

[17] Costa A., Lopez-Villalobos N., Sneddon N.W., Shalloo L., Franzoi M., De Marchi M., Penasa M. (2019). Invited review: Milk lactose—Current status and future challenges in dairy cattle. J. Dairy Sci..

[18] Valde J.P., Hird D.W., Thurmond M.C., Østerås O. (1997). Comparison of ketosis, clinical mastitis, somatic cell count, and reproductive performance between free stall and tie stall barns in Norwegian dairy herds with automatic feeding. Acta Vet. Scand..

[19] Washburn S.P., White S.L., Green J.T., Benson G.A. (2002). Reproduction, mastitis, and body condition of seasonally calved Holstein and Jersey cows in confinement or pasture systems. J. Dairy Sci..

[20] Ferrari F., Cocconi D., Bettini R., Giordano F., Santi P., Tobyn M., Price R., Young P., Caramella C., Colombo P. (2004). The surface roughness of lactose particles can be modulated by wet-smoothing using a high-shear mixer. AAPS PharmSciTech.

[21] Televičius M., Juozaitiene V., Malašauskienė D., Antanaitis R., Rutkauskas A., Urbutis M., Baumgartner W. (2021). Inline Milk Lactose Concentration as Biomarker of the Health Status and Reproductive Success in Dairy Cows. Agriculture.

[22] National Mastitis Council (1987). The Guide to Bovine Mastitis Issues in the Laboratory and in Field.

[23] Ali A.K.A., Shook G.E. (1980). An optimum transformation for somatic cell concentration in milk. J. Dairy Sci..

[24] Argaw A. (2016). Review on epidemiology of clinical and subclinical mastitis on dairy cows. Food Sci. Qual. Manag..

[25] Cobirka M., Tancin V., Slama P. (2020). Epidemiology and Classification of Mastitis. Animals.

[26] Klimienė I., Mockeliūnas R., Butrimaitė-Ambrozevičienė Č., Sakalauskienė R. (2005). The distribution of dairy cow mastitis in Lithuania. Vet. Zootech..

[27] Čereškienė E., Juozaitienė V., Juozaitis A., Černauskienė J., Žymantienė J., Kantautaitė J. (2016). Influence of mastitis agents on milk traits of cows. Vet. Zootech..

[28] Haltia L., Honkanen-Buzalski T., Spiridonova I., Olkonen A., Myllys V. (2006). A study of bovine mastitis, milking procedures and management practices on 25 Estonian dairy herds. Acta Vet. Scand..

[29] Jemeljanovs A., Konosonoka I.H., Bluzmanis J., Ikauniece D. (2008). Changes of mastitis pathogen spectrum in dairy herds of Latvia. Mastitis Control–From Science to Practice.

[30] Vitali A., Bernabucci U., Nardone A., Lacetera N. (2016). Effect of season, month and temperature humidity index on the occurrence of clinical mastitis in dairy heifers. Adv. Anim. Vet. Sci..

[31] Tarazona-Manrique L.E., Villate-Hernández J.R., Andrade-Becerra R.J. (2019). Bacterial and fungal infectious etiology causing mastitis in dairy cows in the highlands of Boyacá (Colombia). Rev. Med. Vet..

[32] Doyle C.J., Gleeson D., O’Toole P.W., Cotter P.D. (2017). Impacts of seasonal housing and teat preparation on raw milk microbiota: A high-throughput sequencing study. Appl. Environ. Microbiol..

[33] Senda A., Nakamura T., Urashima T., Arai I. (2003). Seasonal incidences and populations of yeast in a high-quality raw milk. Anim. Sci. J..

[34] Ranjan R., Gupta M.K., Singh K.K. (2011). Study of bovine mastitis in different climatic conditions in Jharkhand, India. Vet. World.

[35] Da Costa G.M., de Pádua Pereira U., Souza-Dias M.A.G., da Silva N. (2012). Yeast mastitis outbreak in a Brazilian dairy herd. Braz. J. Vet. Res. Anim. Sci..

[36] Krukowski H., Lisowski A. (2010). Occurrence of yeast mastitis in cows in relation to the type of dairy farm, system of milking and maintenance system of cows. Med. Weter..

[37] Keller B., Scheibl P., Bleckmann E., Hoedemaker M. (2000). Differentiation of yeasts in mastitis milk. Mycoses.

[38] Dworecka-Kaszak B., Krutkiewicz A., Szopa D., Kleczkowski M., Biegańska M. (2012). High prevalence of *Candida* yeast in milk samples from cows suffering from mastitis in Poland. Sci. World J..

[39] Masteikienė R. (2006). Maisto produktų mikrobiologija.

[40] Zhang D., Zhang Z., Huang C., Gao X., Wang Z., Liu Y., Tian C., Hong W., Niu S., Liu M. (2017). The phylogenetic group, antimicrobial susceptibility, and virulence genes of *Escherichia coli* from clinical bovine mastitis. J. Dairy Sci..

[41] Petersson-Wolfe C.S., Wolf S.L., Hogan J.S. (2009). Experimental challenge of bovine mammary glands with Enterococcus faecium during early and late lactation. J. Dairy Sci..

[42] Reist M., Erdin D., Von Euw D., Tschuemperlin K., Leuenberger H., Chilliard Y., Hammon H.M., Morel C., Philipona C., Zbinden Y. (2002). Estimation of energy balance at the individual and herd level using blood and milk traits in high-yielding dairy cows. J. Dairy Sci..

[43] Pyörälä S. (2003). Indicators of inflammation in the diagnosis of mastitis. Vet. Res..

[44] Forsbäck L., Lindmark-Månsson H., Andrén A., Åkerstedt M., Andrée L., Svennersten-Sjaunja K. (2010). Day-to-day variation in milk yield and milk composition at the udder-quarter level. J. Dairy Sci..

[45] Gillon A., Bastin C., Soyeurt H., Gengler N. (2010). Genetic parameters of mastitis-correlated milk components in first parity dairy cows. Proceedings of the 9th World Congress on Genetics Applied to Livestock Production.

[46] Haile-Mariam M., Pryce J.E. (2017). Genetic parameters for lactose and its correlation with other milk production traits and fitness traits in pasture-based production systems. J. Dairy Sci..

[47] Ptak E., Brzozowski P., Bieniek J. (2012). Genetic parameters for lactose percentage in the milk of Polish Holstein-Friesians. J. Anim. Feed Sci..

[48] Noro G., González F.H.D., Campos R., Dürr J.W. (2006). Fatores ambientais que afetam a produção e a composição do leite emrebanhos assistidos por cooperativas no Rio Grande do Sul. Rev. Bras. Zootec..

[49] Zanela M.B., Fischer V., Ribeiro M.E.R., Stumpf Junior W., Zanela C., Marques L.T., Martins P.R.G. (2006). Qualidade do leite em sistemas de produção na região Sul do Rio Grande do Sul. Pesqui Agropecu Bras..

[50] Shimkiene A., Juozaitiene V., Juozaitis A., Shimkus A., Zilaitis V., Urbonavicius A. (2009). Relationship between lactose content in cow’s milk with selection attributes and heritability. Vet. Med. Zoot..

[51] Bobbo T., Ruegg P.L., Stocco G., Fiore E., Gianesella M., Morgante M., Pasotto D., Bittante G., Cecchinato A. (2017). Associations between pathogen-specific cases of subclinical mastitis and milk yield, quality, protein composition, and cheese-making traits in dairy cows. J. Dairy Sci..

[52] Coulona J.B., Gasquib P., Barnouin J., Ollier A., Pradel P., Pomiès D. (2002). Effect of mastitis and related-germ on milk yield and composition during naturally-occurring udder infections in dairy cows. Anim. Res..

[53] Kayano M., Itoh M., Kusaba N., Hayashiguchi O., Kida K., Tanaka Y., Kawamoto K., Gröhn Y.T. (2018). Associations of the first occurrence of pathogen-specific clinical mastitis with milk yield and milk composition in dairy cows. J. Dairy Res..

[54] Ianieva O.D., Voronina G.O., Pidgorskyi V.S. (2013). Isolation and characteristics of the lactose-fermenting yeasts *Candida kefyr*. Cytol. Genet..

